# Vitamin D and Retinoic Acid Require Protein Kinase C Activity and Reactive Oxygen Species as Opposing Signals Regulating *PEIG-1*/*GPRC5A* Expression in Caco-2 and T84 Colon Carcinoma Cells

**DOI:** 10.3390/biom15050711

**Published:** 2025-05-13

**Authors:** Pablo A. Iglesias González, Consuelo Mori, Ángel G. Valdivieso, Tomás A. Santa Coloma

**Affiliations:** Laboratory of Cellular and Molecular Biology, Institute for Biomedical Research (BIOMED), School of Medical Sciences, Pontifical Catholic University of Argentina, Alicia Moreau de Justo 1600, Buenos Aires 1107, Argentina; pabloiglesias@uca.edu.ar (P.A.I.G.); consuelo_mori@uca.edu.ar (C.M.); angel_valdivieso@uca.edu.ar (Á.G.V.)

**Keywords:** GPRC5A, PKC, vitamin D, retinoic acid, ATRA, ROS, cancer, chemoprevention

## Abstract

*PEIG-1/GPRC5A* (phorbol ester induced gene-1/G-protein Coupled Receptor Class C Group 5 Member A) was the first identified member of the orphan G protein-coupled receptor family GPRC5. Deregulation of its expression is associated with the development and progression of various types of tumours, particularly colon carcinoma. In this work, we study the effects of vitamin D (VD, cholecalciferol) and retinoic acid (RA) on *GPRC5A* mRNA expression in the colorectal cancer cell lines Caco-2 and T84. Both VD (10 µM) and all-trans retinoic acid (ATRA, atRA, RA) (10 µM) increased *GPRC5A* mRNA levels. Protein kinase C (PKC) inhibition with Gö6983 (10 µM) completely abolished the effects of VD and RA on *GPRC5A* expression. In parallel, VD and RA increased cytosolic and mitochondrial ROS levels (cROS and mtROS). However, the antioxidants NAC (10 mM) and MitoTEMPO (10 µM) raised *GPRC5A* gene expression levels in the presence of VD or RA, suggesting that elevated ROS may inhibit *GPRC5A* expression. In conclusion, both VD and RA stimulate *GPRC5A* expression. The mechanisms involve a common and essential PKC signalling pathway, as Gö6983 inhibited both VD- and RA-induced signalling.

## 1. Introduction

In recent years, the *GPRC5A* gene has received special attention due to its implication in various types of cancer, including lung, breast, colorectal, and prostate cancer [1,2]. *GPRC5A* was the first identified member of a new family of orphan G protein-coupled receptors (GPCRs), known as family C5, which also includes *GPRC5B*, *GPRC5C*, and *GPRC5D*. *GPRC5A* was initially found in our laboratory through differential display as a TPA (12-O-Tetradecanoylphorbol-13-acetate)-induced gene, and named *TIG1* (TPA Induced Gene 1, BE519991). However, since the symbol *TIG1* was already assigned to another gene, it was renamed *PEIG-1* (Phorbol Ester Induced Gene 1, AF506289.1, AAM77594.1) [2,3,4,5,6]. Two years later, also using differential display, Lotan’s laboratory rediscovered this gene as a retinoic acid (RA)-inducible gene (*RAIG-1*) [7]. Its official HGNC (Human Genome Organization, Gene Nomenclature Committee) symbol is now *GPRC5A* (G Protein-Coupled Receptor Class C Group 5 Member A, Gene ID: 9052). Based on its sequence, the other three members of this family were later reported by other authors [8,9].

This gene is predominantly expressed in lung tissue, where it functions as a tumour suppressor gene [10,11]. GPRC5A also exhibits a tumour-suppressive effect in breast cancer cells by inhibiting EGFR (epidermal growth factor receptor) [12]. However, elevated *GPRC5A* expression has been reported in colorectal cancer (CRC), where it promotes tumour progression through VNN1 (Vanin 1)-induced oxidative stress [13] and facilitates cancer cell adaptation to hypoxia through the HIF (hypoxia-inducible factor), GPRC5A, and YAP (YES proto-oncogene 1-associated transcriptional regulator) axis [14], suggesting that *GPRC5A* may also act as a tumour promoter. Additionally, it has been recently linked to gallbladder cancer metastasis through the GPRC5A, JAK2 (Janus kinase 2), STAT3 (signal transducer and activator of transcription 3), and TNS4 (tensin 4) signalling pathways [15].

The phorbol ester TPA induces a strong upregulation of *GPRC5A*, mediated by its binding to PKC [6]. Similarly, the active form of vitamin D (1,25 (OH)_2_-D) directly binds to and modulates PKC activity [16], and the vitamin D receptor (VDR) forms heterodimers with the retinoic acid X receptor (RXR) [17]. In addition, the *GPRC5A* promoter contains three potential retinoic acid response elements (RAREs), two of which correspond to the heterodimer RAR/RXR (retinoic acid receptor/retinoic X receptor) binding sites and one to a VDR (vitamin D receptor)/RXR heterodimeric binding site. However, only the VDR/RXR binding site was found to be a functional RARE in mediating *GPRC5A* induction by RA [18]. RA also induces Ca^2+^-dependent PKC autophosphorylation [19] and modulates PKC-δ (PKC-delta) activity and nuclear translocation, which is an absolute requirement for an effective RA response [20,21]. Since, as mentioned, VDR/RXR was found to be functional in inducing *GPRC5A* expression, the aim of this work was to determine whether VD (cholecalciferol, which is intracellularly converted to its active form 1,25(OH)_2_D or VD_3_) can modulate *GPRC5A* expression in the Caco-2 and T84 colon carcinoma cells, and whether PKC is involved in this regulation. All-trans retinoic acid (abbreviated ATRA or atRA; referred to here as RA for simplicity) was initially used as a positive control for *GPRC5A* stimulation and expression levels. The results show that VD upregulates *GPRC5A*. The effect was similar to that obtained with RA, although with a different response time. Interestingly, PKC inhibition with Gö6983 was later found to block not only the VD response but also strongly inhibit RA signalling. In parallel, increased ROS production induced by VD or RA stimulation partially counteracted *GPRC5A* upregulation, as observed in the presence of the ROS scavengers NAC (N-acetyl-L-cysteine) or MitoTEMPO ([2-[(1-hydroxy-2,2,6,6-tetramethylpiperidin-4-yl)amino]-2-oxoethyl]-triphenylphosphanium;chloride). Thus, VD- and RA-mediated upregulation of GPRC5A involves PKC as a common and key regulatory step upstream of VD and RA nuclear signalling. A preprint corresponding to these results has been published elsewhere [22].

## 2. Materials and Methods

### 2.1. Reagents

Dimethyl sulfoxide (DMSO, culture grade), trypsin (Cat. No. 85450C), N-acetyl-L-cysteine (NAC) (0.5 M stock solution in water pH = 7.4) (Cat. No. A7250), CCCP (carbonyl cyanide m-chlorophenylhydrazone, Cat. Merck No. C2759), and MitoTEMPO (Cat. No. SML0737) were purchased from Sigma Aldrich (St. Louis, MO). MitoSOX 6-(3,8-diamino-6-phenyl-6H-phenanthridin-5-yl)hexyl-triphenylphosphanium iodide (Cat. No. M36008) was obtained from Invitrogen (Carlsbad, CA, USA). 2′,7′-dichlorofluorescein diacetate (DCFH-DA) (Cat. No. D399) and tetramethylrhodamine ethyl ester perchlorate (TMRE) (Cat. No. T669) were purchased from Molecular Probes (Eugene, OR, USA). Retinoic acid (all-trans-retinoic acid, ATRA, atRA or RA, Cat. No. HY-14649), vitamin D (VD) (cholecalciferol, Cat. No. HY-15398), and the PKC inhibitor Gö6983 (3-[1-[3-(dimethylamino)propyl]-5-methoxy-1*H*-indol-3-yl]-4-(1*H*-indol-3-yl)-1*H*-pyrrole-2,5-dione) (Cat. No. HY-13689) were obtained from MedChem Express (MCE, Monmouth Junction, NJ, USA). All other reagents were of analytical grade.

### 2.2. Culture Cells

Human colon adenocarcinoma cell lines T84 (CCL-248) and Caco-2 (HTB-37) were purchased from the American Type Culture Collection (ATCC). These cells were cultured in DMEM/F12 medium (Life Technologies, GIBCO BRL, Rockville, MD, USA), supplemented with 5% FBS (Internegocios S.A., Mercedes, Buenos Aires, Argentina), 100 U/mL penicillin, and 100 µg/mL streptomycin (Life Technologies, GIBCO BRL, Rockville, MD). Cultures were grown at 37 °C in a humidified air atmosphere containing 5% CO_2_ and plated at a density of 20,000 cells/cm^2^. Before treatments, cells were cultured for 24 h in serum-free DMEM/F12 medium. Then, cells were incubated with RA (10 µM) or VD (10 µM) for 4 h and 24 h, respectively. For PKC inhibition and antioxidant scavenger treatments, VD and RA were added simultaneously with the Gö6983 inhibitor (10 μM), MitoTEMPO (10 μM), or NAC (10 mM), without pretreatment.

### 2.3. Quantitative Real-Time RT-PCR (RT-qPCR)

To measure *GPRC5A* mRNA expression levels, RT-qPCR (reverse transcription quantitative real-time polymerase chain reaction) assays were performed using the ΔΔCt method (comparative cycle threshold method), as previously reported [6]. Briefly, total RNA (1–4 µg) from Caco-2 and T84 cells was used for reverse transcription with M-MLV (Moloney Murine Leukemia Virus) reverse transcriptase (RT) (Promega, Cat. No. M1701, 100 U) and specific primers in a final reaction volume of 25 µL, according to the manufacturer’s instructions. The synthesized cDNA was analysed by RT-qPCR using an ABI 7500 real-time PCR system (Applied Biosystems Inc., Foster City, CA, USA). All PCR conditions, primers for *TBP* (*TATA-Box Binding Protein*) and *GPRC5A*, PCR mixes, temperatures, and cycles, were used as previously reported [6]. The RT-qPCR values were expressed as the mean of the relative quantification (RQ) for each replicate ± the standard deviation (mean ± SD (*n*), *n* = 3–5). Results were finally expressed as the mean of three independent experiments ± the standard error of the mean (mean ± SEM (*n*)).

### 2.4. Measurement of Cellular and Mitochondrial Reactive Oxygen Species

Cellular and mitochondrial ROS levels (cROS and mtROS, respectively) were measured using flow cytometry as previously described [23,24], with a few modifications. Briefly, cells were grown in 24-well plates and incubated with RA (10 µM), VD (10 µM), PKC inhibitor Gö6983 (10 µM), MitoTEMPO (10 µM), NAC (10 mM) or with equivalent amounts of vehicle (DMSO, culture grade, final concentration 0.1%) for 4 h or 24 h in serum-free DMEM/F12, as indicated in the figures. To measure cROS levels, cells were incubated with DCFH-DA (10 µM) (Ex/Em 510/540 nm, FL1 channel) in serum-free DMEM/F12, at 37 °C in a 5% CO_2_/air incubator for 30 min. To measure mtROS levels, cells were incubated with MitoSOX (5 µM) (Ex/Em 510/580 nm, FL2 channel) at 37 °C in a 5% CO_2_/air incubator for 10 min. Then, cells were washed three times in PBS buffer and harvested with trypsin incubation (0.25% trypsin, 0.02% EDTA in PBS). Cells were collected by centrifugation at 400× *g* for 5 min, resuspended in 200 µL Hank’s buffer, and analysed by flow cytometry (Accuri, BD Biosciences, San José, CA, USA). The mean fluorescence intensity (MFI) values of the FL1 and FL2 channels were used to quantify cROS and mtROS, respectively. Data were normalized using the values for untreated cells (controls) set to 1 unit.

### 2.5. Measurement of Mitochondrial Membrane Potential (Ψm)

Changes in the Ψm were evaluated by flow cytometry using the TMRE probe (Ex/Em: 549/573 nm) as previously reported by Crowley et al. [25]. Briefly, cells were grown in 24-well plates and incubated with RA (10 µM), VD (10 µM), or vehicle (DMSO, culture grade, final concentration 0.1%) for 4 h or 24 h in serum-free DMEM/F12. As a control of Ψm modulation, a group of cells was treated with CCCP (20 μM), an uncoupler of mitochondrial oxidative phosphorylation. Then, cells were washed with PBS twice and incubated in DMEM/F12 medium containing 40 nM of TMRE at 37 °C for 20 min, washed three times with PBS, and harvested with trypsin incubation (0.25% trypsin, 0.02% EDTA in PBS). Lastly, cells were resuspended in 300 µL Hank’s buffer and analysed by flow cytometry. TMRE fluorescent events were detected on the FL2 channel. Data were normalized using the values for untreated cells (control) set to 1 unit.

### 2.6. Statistics

The assays were performed in at least triplicate, and the experiments were independently repeated at least three times (*n* = 3). The final RT-qPCR quantification values were obtained as the means of the relative quantification (RQ) values for each independent experiment (*n* = 3). One-way ANOVA and Tukey’s post hoc test were applied (GraphPad Prism 10.0) to determine significant differences when more than two factors were compared (*p* < 0.05). Student’s *t* tests were performed to detect significant differences when only two factors were compared. All values are shown as mean ± SEM (*n*); the number of biological inter-assay replicates (*n*) is indicated in each case. Bar graphs include the individual means of each independent experiment as open dots and the averaged means as bars; SEMs are shown as error bars [26].

## 3. Results

### 3.1. Effects of VD and RA on GPRC5A Expression

In this study, T84 cells were used since we discovered and cloned *GPRC5A* in these cells under TPA stimulation, with maximal response at 4 h [3]. Caco-2 cells were added to further confirm the results. Later studies on *GPRC5A* gene regulation have shown that retinoic acid (RA) also acts as a potent inducer of its transcription in the head and neck squamous carcinoma cell line UMSCC-22B [7]. Therefore, stimulation of *GPRC5A* expression by RA was used as a positive control for T84 and Caco-2 cells and to provide a reference for comparing the relative *GPRC5A* response to RA and VD. To find the optimal conditions, Caco-2 and T84 cells were treated with different concentrations of RA (ATRA, 0, 0.1, 1, or 10 µM) for 4 h, which was the time for maximal GPRC5A expression under TPA stimulation. Separately, T84 and Caco-2 cells were treated with VD (0, 1, 5, 10, 20, or 40 µM) for a longer time (24 h), since VD needed time for conversion to VD_3_. After incubation, the *GPRC5A* expression levels were measured using RT-qPCR.

**Figure 1:**
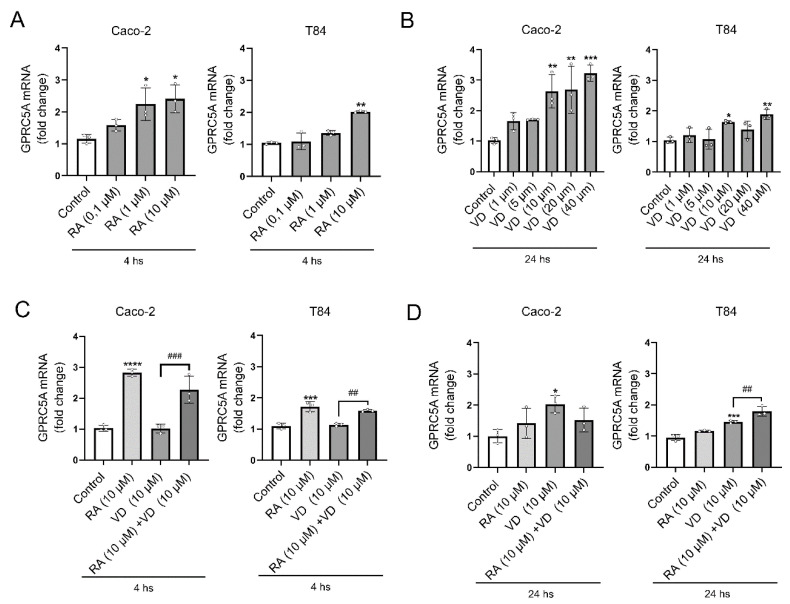
VD and RA increase *GPRC5A* mRNA expression. (**A**) *GPRC5A* mRNA expression levels in Caco-2 and T84 cell lines, following RA (0.1–10 µM) treatment for 4 h. (**B**) *GPRC5A* mRNA expression levels in Caco-2 and T84 cell lines following VD (1–40 µM) treatment for 24 h (**C**) *GPRC5A* mRNA expression levels after combined RA and VD treatment for 4 or (**D**) 24 h. Data are expressed as mean ± SEM (*n*) for three independent experiments (*n* = 3). Each dot represents the mean value of each independent experiment. Statistical analyses were performed using ANOVA followed by Tukey’s post hoc tests. (* *p* < 0.05; ** *p* < 0.01; *** *p* < 0.001; **** *p* <0.0001; ## *p* < 0.01, ### *p* < 0.001). All figures regarding expression levels represent fold changes compared to control values.

As shown in Figure 1A, left panel, the *GPRC5A* expression was significantly induced with RA 1 µM (2.24 ± 0.51 (*n* = 3), *p* < 0.05) and RA 10 µM (2.40 ± 0.44 (*n* = 3), *p* < 0.05; all *GPRC5A* mRNA results are expressed as fold changes relative to control values, which are taken as 1 unit) in Caco-2 cells. A similar trend was observed in T84 cells, although in these cells *GPRC5A* levels were significantly increased only at 10 µM RA (2.01 ± 0.03 (*n* = 3), *p* < 0.01) (Figure 1A, right panel). Based on these results, a concentration of 10 µM RA (for 4 h) was selected for further experiments.

On the other hand VD induced a significant increase in *GPRC5A* mRNA levels in Caco-2 cells at concentrations of 10 µM for 24 h (2.63 ± 0.55 (*n* = 3), *p* < 0.01), 20 µM (2.68 ± 0.77 (*n* = 3), *p* < 0.01), and 40 µM (3.22 ± 0.26 (*n* = 3), *p* < 0.001), in a dose–response manner (Figure 1B, left panel). Similar results were obtained with T84 cells after 24 h of VD incubation (Figure 1B, right panel), although to a much lesser extent than in Caco-2 cells. At 4 h, VD 10 µM showed no effects on GPRC5A expression levels (Figure 1C) in both Caco-2 and T84 cells. The response time to VD is different compared to RA (24 h vs. 4 h). VD needs time to be converted into VD_3_ [27]; the enzymes involved and their expression levels in Caco-2 and T84 cells are shown in Table S1.

The potential additive or synergistic effects of VD and RA on the regulation of the *GPRC5A* expression were also explored at 4 h or 24 h. As shown in Figure 1C, there were no observed additive or synergistic effects when cells were treated with VD and RA for 4 h in both T84 and Caco-2 cells, but rather a slight decrease in *GPRC5A* expression. After 24 h, as shown in Figure 1D, right panel, the combined treatment resulted in a small but significant increase in *GPRC5A* expression in T84 cells, compared to each treatment separately (RA: 1.16 ± 0.01, ns; VD: 1.45 ± 0.04, *p* < 0.001; RA + VD: 1.79 ± 0.15 (*n* = 3), *p* < 0.0001). Instead, in Caco-2 cells, the effect of VD was slightly attenuated by RA (RA: 1.41 ± 0.48, ns; VD: 2.02 ± 0.27, *p* < 0.05; RA + VD: 1.52 ± 0.38 (*n* = 3), ns) (Figure 1D, left panel). In conclusion, in these conditions, only a slight additive effect was observed in T84 cells after 24 h of incubation with VD and RA (Figure 1D, right panel). The *GPRC5A* response to RA is reduced after 24 h of incubation compared to 4 h. This is consistent with previous results from TPA-stimulated cells, showing maximal TPA stimulation at 4 h and lower levels at 6 h [6] and 24 h [4]. Further studies are needed to better understand the potential combined effects of VD and RA on *GPRC5A* expression.

### 3.2. PKC Inhibition with Gö6983 Completely Blocks VD Effects on GPRC5A mRNA Expression

**Figure 2:**
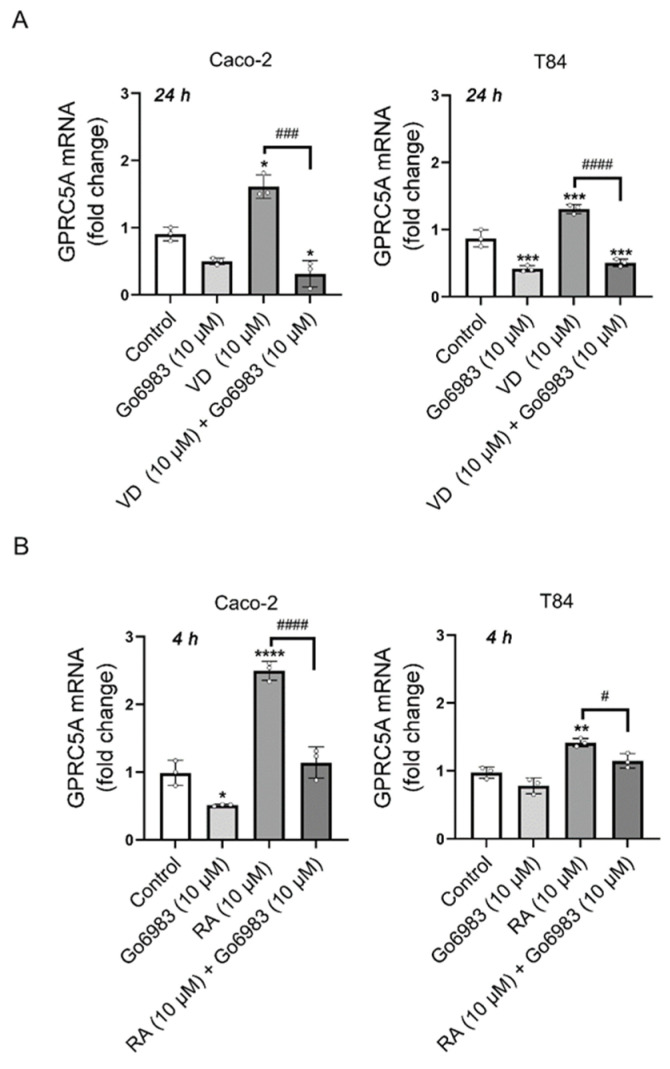
Modulation of GPRC5A gene expression. (**A**) Changes in GPRC5A mRNA expression levels in Caco-2 (left panel) and T84 (right panel) cell lines, in the presence or absence of VD (10 µM) and Gö6983 (10 µM), for 24 h. (**B**) Changes in GPRC5A mRNA expression levels in Caco-2 and T84 cell lines, in the presence or absence of RA (10 µM) and Gö6983 (10 µM), for 4 h (* *p* < 0.05; ** *p* < 0.01; *** *p* < 0.001; **** *p* < 0.0001; # *p* < 0.05; ### *p* < 0.001; #### *p* < 0.0001).

Previously, we reported the involvement of PKC in the upregulation of *GPRC5A* [2,3,4,5,6]. This became evident when T84 cells were treated with the phorbol ester TPA (PMA), highlighting the role of PKC in the induction of *GPRC5A*. To assess the potential involvement of PKC in regulating *GPRC5A* levels induced by VD or RA, we employed the pan-PKC inhibitor Gö6983 at a concentration of 10 μM [28], as previously reported for the effects of TPA on *GPRC5A* [6]. As shown in Figure 2, Gö6983 effectively blocked the induction of *GPRC5A* expression by VD (Caco-2: 1.61 ± 0.17 vs. 0.31 ± 0.19 (*n* = 3), *p* < 0.001; T84: 1.30 ± 0.06 vs. 0.50 ± 0.05 (*n* = 3), *p* < 0.0001) (Figure 2A). Similar results were observed for RA, where levels of *GPRC5A* returned control values when the PKC pathway was inhibited (Figure 2B) (Caco-2: 2.49 ± 0.13 vs. 1.14 ± 0.23 (*n* = 3), *p* < 0.0001; T84: 1.41 ± 0.06 vs. 1.14 ± 0.10 (*n* = 3), *p* < 0.05). The results show that both signalling pathways, RA and VD, converge in PKC. The results also suggest that the nuclear signalling that determines the *GPRC5A* response to VD and RA is not functional when PKC activity is inhibited.

### 3.3. Effects of VD and RA on ROS Levels and Mitochondrial Membrane Potential (Ψm) in Caco-2 and T84 Cells

It has been reported that VD activates PKC in Caco-2 cells [29]. Also, both VD [30] and RA [31] modulate ROS levels. In particular, increased mtROS generation is mediated by PKC-δ (isoform delta) in hepatocarcinoma cell lines treated with TPA [32], and TPA produces a strong upregulation of *GPRC5A* through PKC [5,6]. Therefore, we measured the cytosolic (cROS) and mitochondrial ROS (mtROS) levels in Caco-2 and T84 cells treated with VD or RA and studied their effects on GPRC5A expression.

**Figure 3:**
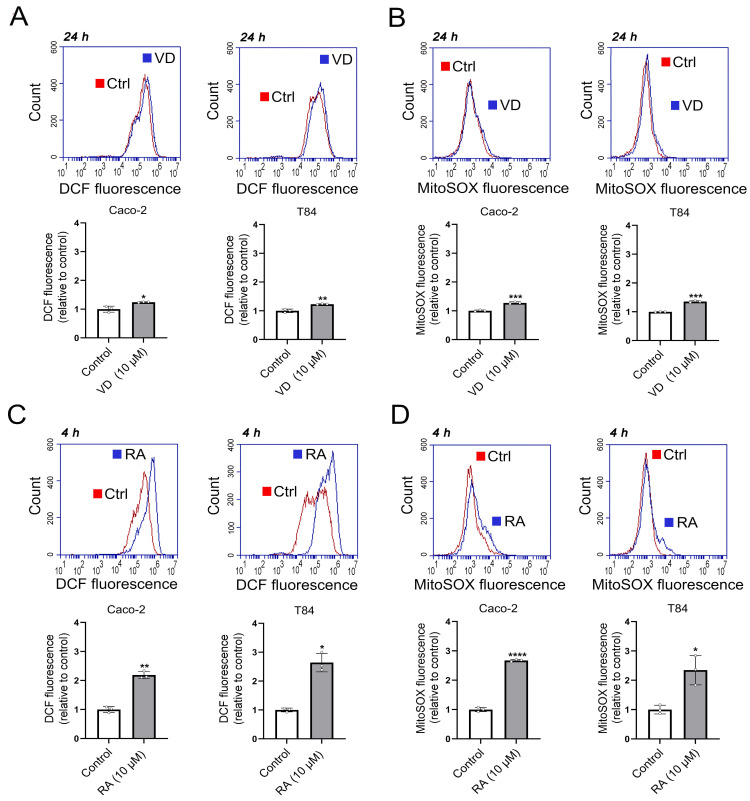
VD and RA induce changes in ROS levels. Changes in cytoplasmic (DCFH) and mitochondrial ROS (MitoSOX) fluorescence levels in Caco-2 cells (left panels) and T84 cells (right panels) treated with vitamin D (VD) or retinoic acid (RA). (**A**) cROS levels in the presence of VD (10 µM) for 24 h. (**B**) mtROS levels in the presence of VD (10 µM) for 24 h. (**C**) cROS levels in the presence of RA (10 µM) for 4 h. (**D**) mtROS levels in the presence of RA (10 µM) for 4 h (* *p* < 0.05; ** *p* < 0.01; *** *p* < 0.001; **** *p* < 0.0001).

The cROS and mtROS were measured by flow cytometry using the DCFH-DA and MitoSOX fluorescent probes, respectively. As shown in Figure 3A, treatment with VD (10 μM) for 24 h led to a small but significant increase in cROS levels in Caco-2 cells (1.24 ± 0.01 (*n* = 3), *p* < 0.05) and T84 cells (1.23 ± 0.01 (*n* = 3), *p* < 0.01). These results were accompanied by a similar small but significant increase in mtROS levels in Caco-2 (1.27 ± 0.03 (*n* = 3), *p* < 0.001) and T84 cells (1.35 ± 0.03 (*n* = 3), *p* < 0.001) (Figure 3B). On the other hand, RA treatment (10 μM) for 4 h significantly elevated cROS levels in Caco-2 (2.18 ± 0.12 (*n* = 3), *p* < 0.01) and T84 cells (2.64 ± 0.32 (*n* = 3), *p* < 0.05) (Figure 3C). RA treatment also increased mtROS levels by more than two-fold (Caco-2: 2.67 ± 0.04 (*n* = 3), *p* < 0.0001; T84: 2.34 ± 0.5 (*n* = 3), *p* < 0.05) (Figure 3D). Supplementary Figures S1 and S2 show the corresponding histograms and dot plots. The effects of RA in cROS and mtROS accumulation were stronger (~two-fold) than those obtained with VD.

To determine whether the VD and RA, both at 10 µM, affected the general mitochondrial function, we evaluated the mitochondrial membrane potential (Ψm) using TMRE and flow cytometry [25]. VD did not produce any significant changes in the membrane potential, in either Caco-2 or T84 cells (Figure S3A). However, TMRE fluorescence was diminished in Caco-2 cells after RA treatment (0.69 ± 0.04 (*n* = 3), *p* < 0.05) (Figure S3B), reflecting a reduced Ψm, in agreement with the increased ROS production seen with RA (Figure 3D) compared to VD (Figure 3B).

### 3.4. Effects of the ROS Scavengers NAC or MitoTEMPO on GPRC5A mRNA Expression After VD or RA Treatment

**Figure 4:**
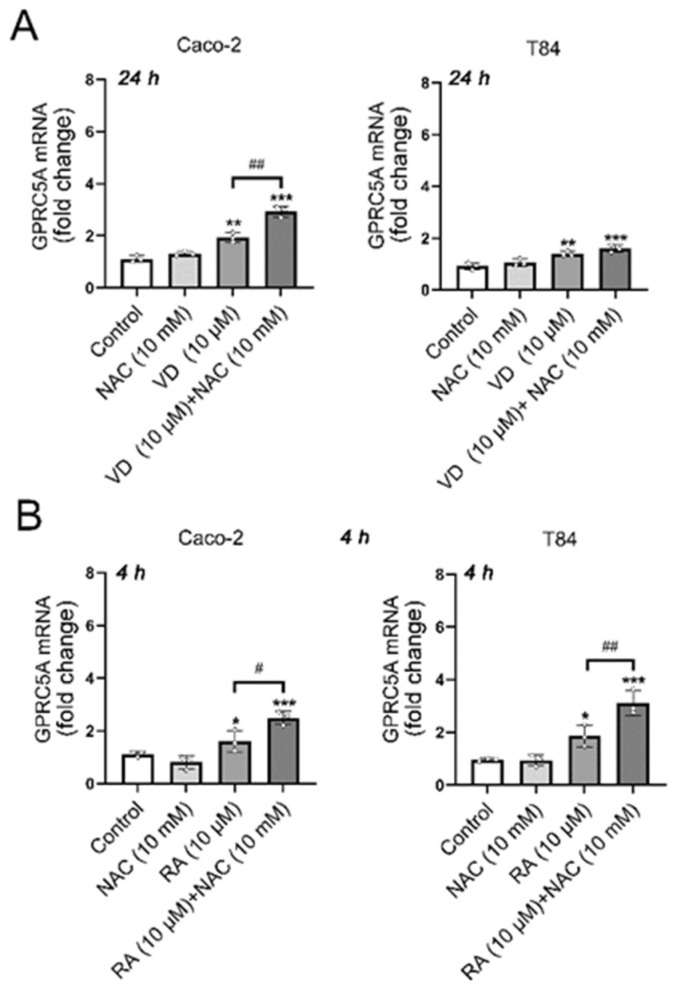

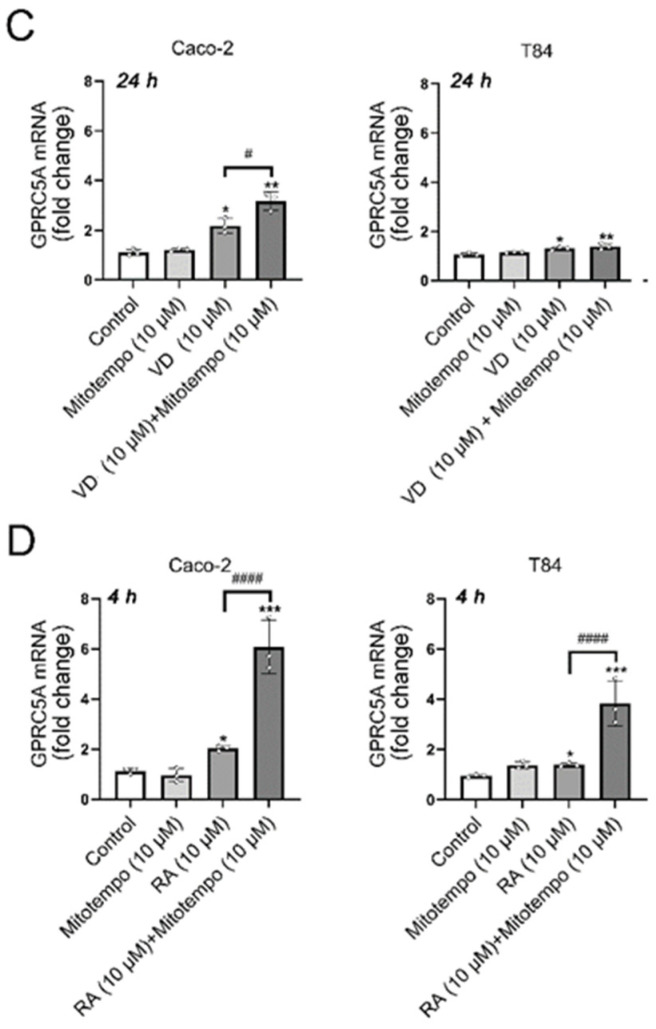
Modulation of GPRC5A gene expression in the presence of ROS scavengers. (**A**) GPRC5A mRNA expression levels in Caco-2 and T84 cell lines, in the presence/absence of VD (10 µM) and NAC (10 mM). (**B**) GPRC5A mRNA expression levels in Caco-2 and T84 cell lines, in the presence/absence of RA (10 µM) and NAC (10 mM). (**C**) GPRC5A mRNA expression levels in Caco-2 and T84 cell lines, in the presence/absence of VD (10 µM) and MitoTEMPO (10 µM). (**D**) GPRC5A mRNA expression levels in Caco-2 and T84 cell lines, in the presence/absence of RA (10 µM) and MitoTEMPO (10 µM). The strongest response was observed with MitoTEMPO and RA (* *p* < 0.05; ** *p* < 0.01; *** *p* < 0.001; # *p* < 0.05; ## *p* < 0.01; #### *p* < 0.0001).

**Figure 5:**
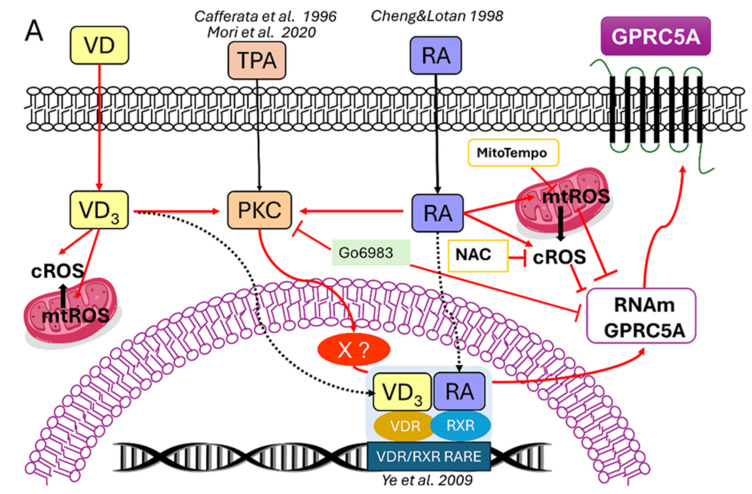
Schematic diagram illustrating the *GPRC5A* regulation by VD and RA. In earlier studies, TPA [3,4,5,6,36] and RA [7,19,37] have been shown to upregulate *GPRC5A* expression. TPA upregulation was mediated by PKC through a yet-to-be-identified transcription factor X [5,6]. *GPRC5A* upregulation by RA was mediated through the heterodimeric transcription factor VDR/RXR [18]. In this work (red arrows), we show that VD also induces *GPRC5A* mRNA expression. The PKC inhibitor Gö6983 strongly inhibited the VD and RA upregulation of *GPRC5A*. It has been reported by other authors that both RA [19,20] and VD [22] might act either directly on PKC (non-genomic effects) or through their nuclear receptors (genomic effects) [19]. In addition, RA induces Ca^2+^-dependent PKC autophosphorylation [19] and modulates PKC activity (PKC-δ) and nuclear translocation, which is essential for an effective RA response [20,21]. It is not known yet for *GPRC5A* if X in the figure represents PKC-δ, another isoform, or a different effector. In addition, VDR/RXR has been reported to be present and active in Caco-2 cells [38,39]; however, direct evidence for VDR/RXR involvement in the VD_3_ → GPRC5A upregulation remains to be obtained.

To determine whether the increased ROS levels following VD or RA treatment affected *GPRC5A* expression, two ROS scavengers were used: N-acetylcysteine (NAC) and MitoTEMPO, in the presence or absence of VD and RA. NAC, a cytosolic ROS (cROS) scavenger, reacts with OH^•^ and H_2_O_2_ and has the capacity to restore GSH levels [33]. Additionally, although to a lesser extent, it can also react with mitochondrial superoxide [34]. On the other hand, MitoTEMPO serves as a mitochondrial-targeted scavenger for superoxide [35]. The presence of NAC (10 mM) or MitoTEMPO (10 µM) did not alter the basal expression of GPRC5A in either Caco-2 or T84 cell lines. However, the induction of GPRC5A expression caused by VD or RA was significantly augmented in the presence of these scavengers in both cell lines (Figure 4). Notably, treatment of Caco-2 cells with MitoTEMPO and RA (10 µM) (Figure 4D, left panel) produced the strongest response: a six-fold stimulation of *GPRC5A* expression. Thus, the increased ROS levels obtained after VD or RA treatments rather counteract the effects of VD or RA on *GPRC5A* expression. A graphical abstract of the results obtained here is shown in Figure 5. Red arrows illustrate the present findings.

## 4. Discussion

The results obtained here show that vitamin D (VD) induced upregulation of *GPRC5A* expression in Caco-2 and T84 cells, with higher levels of expression seen in Caco-2 cells. The mechanism of upregulation involved PKC, since the pharmacological inhibition of PKC with Gö6983 completely blocked the VD effects. Equivalent results were obtained with RA stimulation. These results agree with earlier work reporting direct effects of VD [21] and RA [19,20] on PKC activity [20] and with the PKC-dependent upregulation of *GPRC5A* after TPA stimulation [3,4,5,6]. The inhibition of the RA response with Gö6983 also agrees with the notion that activation of PKC-δ (isoform delta) and its translocation to the nucleus is essential for RA response through retinoic acid receptors and their retinoic acid response elements (RAREs) [20]. However, the results obtained with the pharmacological inhibitor Gö6983 should be interpreted with caution, as they are not conclusive due to potential off-target effects commonly associated with pharmacological inhibitors. Further studies will aim to validate these findings using alternative approaches to inhibit PKC, including other pharmacological inhibitors with distinct mechanisms of action and RNA interference strategies. These approaches will help assess their effectiveness in blocking PKC autophosphorylation and downstream effects on PKC substrates. Additionally, it will be necessary to identify the PKC-activated signalling pathway(s) that lead to increased GPRC5A mRNA and protein expression (or increased degradation). Determining which PKC isoform(s) are involved in each context (VD or RA) and analysing the promoter structure and its regulatory mechanisms will also be essential. Finally, the pathophysiological significance of these findings must be established.

In parallel with the augmented *GPRC5A* expression under VD or RA stimulation, there was a significant rise in both cROS and mtROS production, with RA exerting a more pronounced effect than VD, whose effects were modest. To determine whether the increased ROS levels affect *GPRC5A* expression, the cells were stimulated in the presence of NAC or MitoTEMPO. Interestingly, the presence of these ROS scavengers rather enhanced the induction of *GPRC5A* expression caused by VD and RA. These results suggest that ROS limit the induction effect caused by VD and RA on GPRC5A expression. The underlying mechanism(s) might be multiple and remain to be elucidated. However, they are consistent with earlier findings showing an increased NF-κB activation induced by ROS through an IL-1β autocrine loop in Caco-2 cells [40,41,42,43], and with the negative effect of NF-κB (Nuclear Factor kappa-light-chain-enhancer of activated B cells) activation on *GPRC5A* expression [44].

While this work was in progress [22], Sampei et al. found that mouse *Gprc5a* expression was induced additively by co-treatment with PTH and calcitriol or retinoic acid in MC3T3-E1 mouse cells [45]. They suggested that *Gprc5a* is a PTH (Parathyroid Hormone)-dependent gene that inhibits cell proliferation and osteoblast differentiation, and might be a suitable candidate as a drug target for osteoporosis.

On the other hand, considering that VD and RA upregulate *GPRC5A* and, in turn, this receptor negatively regulates EGFR [11,12,46], it might be part of the mechanism of RA chemoprevention described by Anita Roberts and Michael Sporn in the 1980s [47]; it may also be involved in the chemoprevention mechanism later reported for VD [48,49]. The controversial results obtained in RA and VD cancer chemoprevention [49] might, therefore, result from the balance between the multiple parallel and often opposing signalling pathways activated by *GPRC5A* in different tumours (e.g., EGFR inhibition [46] vs. HIF-[1,2GPRC5A-YAP [14], or GPRC5A-JAK2-STAT3-TNS4 [15]). Since the mechanisms of *GPRC5A* signalling and the relative relevance of their effects are not fully understood, caution should be exercised when using VD or RA in the chemoprevention or treatment of the different diseases in which *GPRC5A* is involved [1,2].

In summary, VD upregulates *GPRC5A* expression. The signalling pathways of VD and RA converge at PKC, as pharmacological inhibition of this kinase with Gö6983 completely blocks *GPRC5A* upregulation by either VD or RA. Thus, PKC appears to function as an essential non-genomic effector in both pathways, acting upstream of their transcription factors.

## Data Availability

The original contributions presented in this study are included in the article/Supplementary Material. Further inquiries can be directed to the corresponding author.

## Supplementary Materials

The following supporting information can be downloaded at: https://www.mdpi.com/article/10.3390/biom15050711/s1, Table S1. Vitamin D 25-hydroxylase (CYP2R1) and 1-α-hydroxylase (CYP27B1) mRNA levels in Caco-2 and T84 cells; Figure S1. Analysis of ROS levels in Caco-2 and T84 cells treated with vitamin D by flow cytometry. (A, B); Figure S2. Analysis of ROS levels in Caco-2 and T84 cells treated with retinoic acid by flow cytometry; Figure S3. Effects of vitamin D and RA (all-trans retinoic acid) on mitochondrial membrane potential (Ψm).
