# Supplementary material for: Vitamin D and Retinoic Acid Require Protein Kinase C Activity and Reactive Oxygen Species as Opposing Signals Regulating PEIG-1/GPRC5A Expression in Caco-2 and T84 Colon Carcinoma Cells
Source: Biomolecules. 2025 May 13;15(5):711. doi: 10.3390/biom15050711 (PMC12108993; doi:10.3390/biom15050711)
Supplement: Supplementary file 1 [file biomolecules-15-00711-s001.zip › biomolecules-3529299-supplementary.pdf]

Supplementary Results

Vitamin D and Retinoic Acid induce PKC activity and reactive oxygen species (ROS) as opposed signals regulating *PEIG-1/GPRC5A* expression in Caco-2 and T84 Colon Carcinoma Cells

Pablo A. Iglesias González, Consuelo Mori, Ángel G. Valdivieso and

Tomás A. Santa Coloma *

Laboratory of Cellular and Molecular Biology, Institute for Biomedical Research (BIOMED), School of Medical Sciences, Pontifical Catholic University of Argentina (UCA), and the National Scientific and Technical Research Council of Argentina (CONICET), Buenos Aires, Argentina.

* Correspondence: tomas_santacoloma@uca.edu.ar or tsantacoloma@gmail.com;

Tel: +5411-3056-9438

Table S1: Vitamin D 25-hydroxylase (CYP2R1) and 1-α-hydroxylase (CYP27B1) mRNA levels in Caco-2 and T84 cells. The vitamin D (VD) active compound is 1,25(OH)$_2$D (calcitriol or VD$_3$). Caco-2 cells have the enzymes needed to convert VD (cholecalciferol) to 1,25(OH)$_2$D (calcitriol, VD$_3$), which are vitamin D 25-hydroxylase (CYP2R1) and 1-α-hydroxylase (CYP27B1) [27]. On the other hand, T84 cells express more 25-hydroxylase (CYP2R1) mRNA and less 1-α-hydroxylase (CYP27B1) mRNA than Caco-2 cells (proteinatlas.org). nTPM indicate normalized transcript per million. The low level of 1-α-hydroxylase mRNA expression in T84 cells agrees with the lower response of T84 cells to cholecalciferol. Caco-2 control cells have 1.7 fmol/10^6 cells of 1,25(OH)$_2$D$_3$ and these levels increase to 7.8 fmol/10^6 cells after UVB irradiation and conversion of 7-dehydrocholesterol (7-DHC, provitamin D$_3$) to 1,25(OH)$_2$D$_3$ (VD$_3$), showing that Caco-2 cells are able to produce VD$_3$ from 7-DHC [27]. In comparison THP-1 basal cells have 3.9 fmol/10^6 cells and increase to 6.3 fmol/10^6 cells after UVB irradiation[27]. In T84 cells basal or UVB stimulated levels are unknown. Caco-2 and T84 cells require time to convert cholecalciferol (vitamin D, VD) into its active form, calcitriol (1,25(OH)$_2$D, VD$_3$). Caco-2 cells express the enzymes necessary for this conversion—vitamin D 25-hydroxylase (CYP2R1) and 1α-hydroxylase (CYP27B1) [27]. Although T84 cells show a higher mRNA expression of CYP2R1, they exhibit significantly lower levels of CYP27B1 mRNA compared to Caco-2 cells (Table S1). This reduced expression of CYP27B1 in T84 cells is consistent with the weaker GPRC5A response to VD observed in these cells, relative to Caco-2 cells (Fig. 1B at 24 h, and Fig. 1C at 4 h). Based on these findings, an incubation time of 24 h and a VD concentration of 10 μM—conditions that yielded an intermediate level of regulation—were selected for subsequent experiments.

Cells	vitamin D 25-hydroxylase (CYP2R1)<br>Units in nTPM<br>Data from proteinatlas.org	1-α-hydroxylase (CYP27B1)<br>Units in nTPM<br>Data from proteinatlas.org	1,25(OH)$_2$D$_3$ Content (fmol/10^6 cells). Data from [27]
Caco-2	4.9	2.0	1.7
T84	8.8	0.1	nd

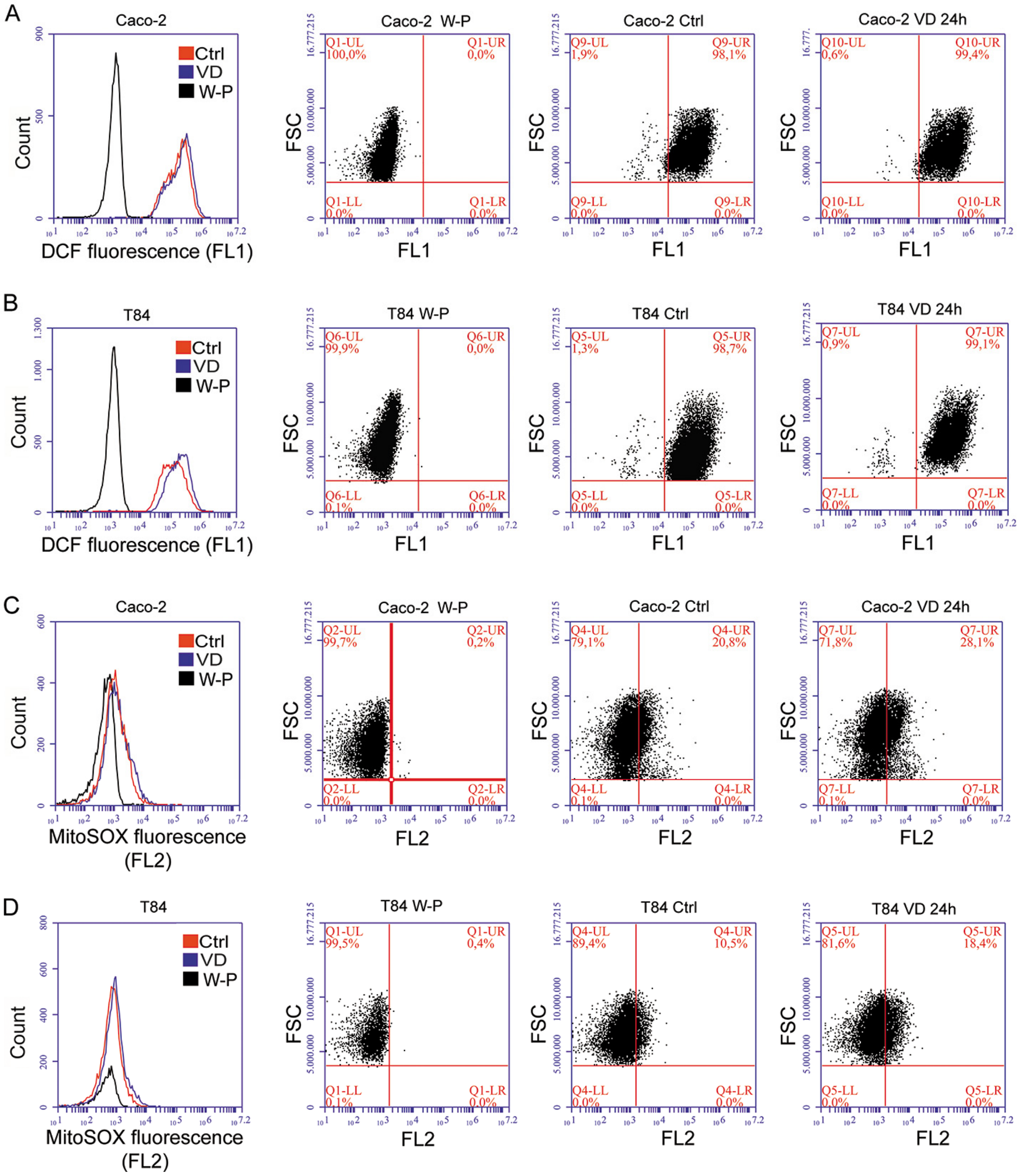

Figure S1. Analysis of ROS levels in Caco-2 and T84 cells treated with vitamin D by flow cytometry. (A, B) Cytoplasmic ROS (cROS) levels measured by DCF fluorescence in Caco-2 **(A)** and T84 **(B)** cells after treatment with vitamin D (VD, 10 µM) for 24 h. **(C, D)** Mitochondrial ROS (mtROS) levels measured by MitoSOX fluorescence in Caco-2 **(C)** and T84 **(D)** cells after treatment with VD (10 µM) for 24 h. Histograms represent fluorescence intensity distributions (Count stand for the frequency of events counted), while Dot Plots show forward scatter (FSC) values versus fluorescence intensity (FL1 for cROS and FL2 for mtROS) under different conditions: without probe (W-P, black line), untreated control (Ctrl, red line), and VD 24 h (blue line). Quadrant analysis indicates the percentage of positive cells (or events) in each condition.

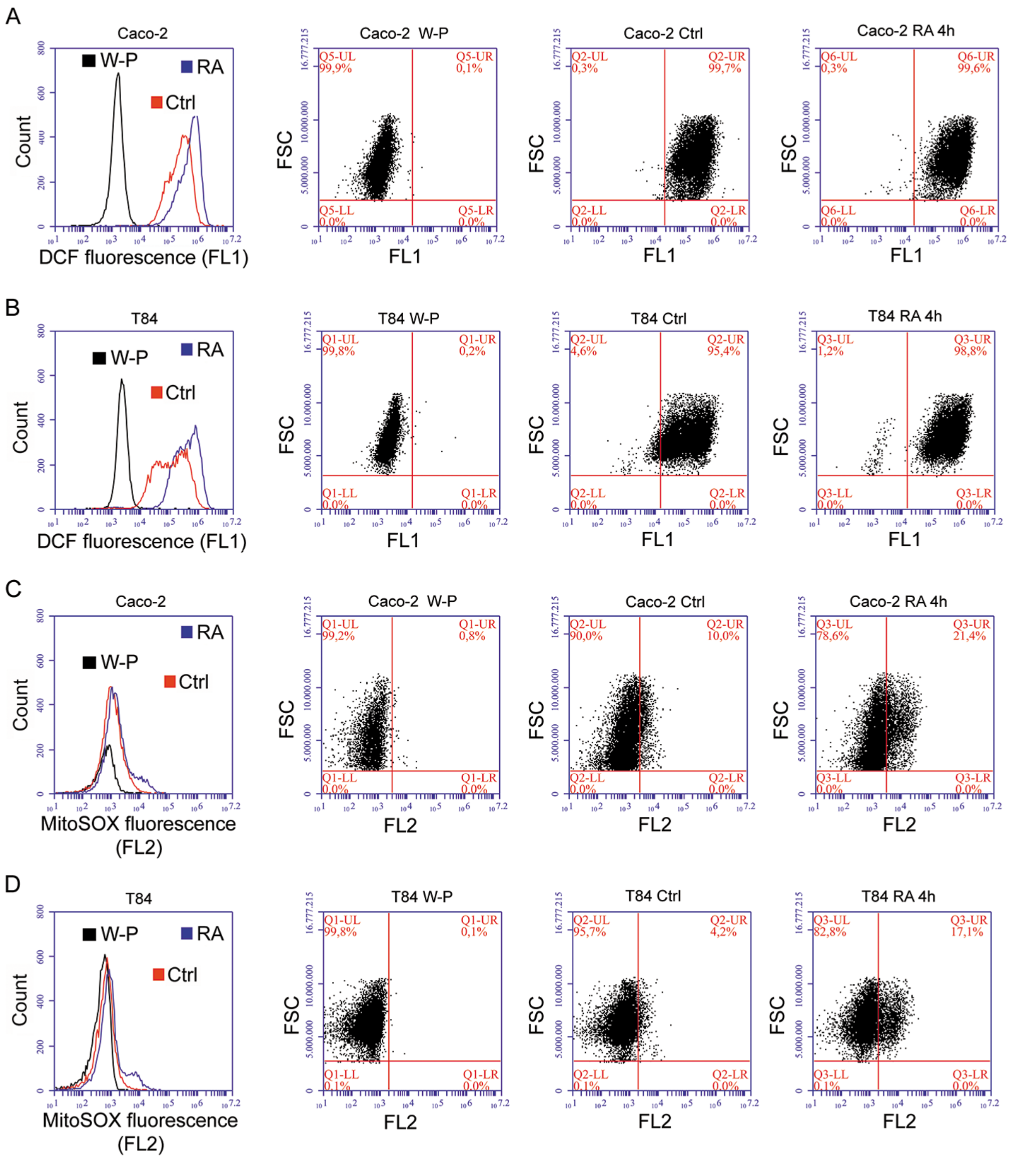

Figure S2. Analysis of ROS levels in Caco-2 and T84 cells treated with retinoic acid by flow cytometry. (A, B) Cytoplasmic ROS (cROS) levels measured by DCF fluorescence in Caco-2 **(A)** and T84 **(B)** cells after treatment with retinoic acid (RA, 10 µM) for 4 h. **(C, D)** Mitochondrial ROS (mtROS) levels measured by MitoSOX fluorescence in Caco-2 **(C)** and T84 **(D)** cells after treatment with RA (10 µM) for 4 h. Histograms represent fluorescence intensity distributions, while scatter plots show forward scatter (FSC) versus fluorescence (FL1 for cROS and FL2 for mtROS) under different conditions: without probe (W-P, black line), untreated control (Ctrl, red line), and RA 4 h (blue line). Quadrant analysis shows the percentage of positive cells in each condition.

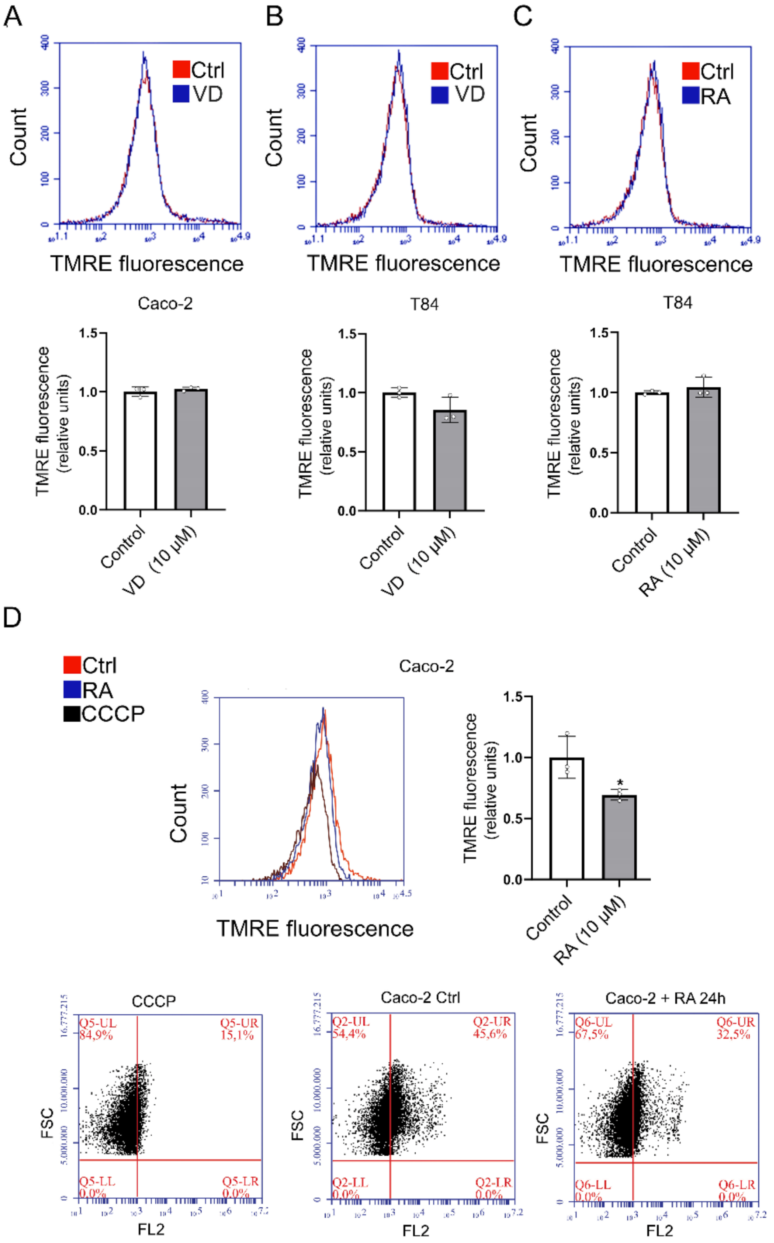

Figure S3. Effects of vitamin D and RA (all-trans retinoic acid) on mitochondrial membrane potential (Ψm). (A–C) TMRE fluorescence analysis in Caco-2 **(A)** and T84 **(B, C)** cells after treatment with vitamin D (VD, 10 µM) for 24 h or retinoic acid (RA, 10 µM) for 4. Histograms and bar graphs show no significant changes in Ψm compared to the control condition. **(D)** TMRE fluorescence in Caco-2 cells after RA treatment (10 µM, 4 h) showing a significant reduction (* $p < 0.05$ vs. control) in Ψm. CCCP (20 µM) was used as a positive control for mitochondrial depolarization. Histograms show a shift in TMRE fluorescence, and scatter plots (FSC vs. FL2) illustrate the distribution of fluorescence intensity across different conditions. Quadrant Q6-UR analysis highlights the decrease in TMRE-positive cells following RA treatment. A significant Ψm

change was only observed in Caco-2 cells treated with RA. VD treatments did not change Ψm significantly.

References:

27. Vantieghem, K.; Overbergh, L.; Carmeliet, G.; De Haes, P.; Bouillon, R.; Segaert, S. UVB-induced 1,25(OH)2D3 production and vitamin D activity in intestinal CaCo-2 cells and in THP-1 macrophages pretreated with a sterol Delta7-reductase inhibitor. *J. Cell. Biochem.* **2006**, *99*, 229-240. doi: 10.1002/jcb.20910.
